# Calcitonin Gene-Related Peptide-Induced Calcium Alginate Gel Combined with Adipose-Derived Stem Cells Differentiating to Osteoblasts

**DOI:** 10.1007/s12013-015-0630-8

**Published:** 2015-05-24

**Authors:** Chang-zhi Huang, Xiao-ning Yang, Da-cheng Liu, Yi-gong Sun, Xing-ming Dai

**Affiliations:** 1 Department of Orthopedics, Ningde Hospital Affiliated to Fujian Medical University, Ningde, 352100 Fujian China; 2 Department of Orthopedics, The First People’s Hospital of Xuzhou, Xuzhou Medical College, Xuzhou, 221000 Jiangsu China

**Keywords:** Calcitonin gene-related peptide, Adipose-derived stem cells, Osteogenic differentiation, Calcium alginate gel, Three-dimensional cultures

## Abstract

Calcitonin gene-related peptide (CGRP) has been confirmed with induction osteoblastic differentiation, but if it can make the three-dimensional culture of adipose-derived stem cells (ADSCs) to the osteoblastic differentiation, thus constructing tissue-engineered bone rare reports. To investigate the feasibility of exogenous CGRP-induced calcium alginate gel combined with ADSCs from rabbits in three-dimensional condition to construct tissue-engineered bone. ADSCs were obtained by collagenase I digestion of the subcutaneous adipose tissue of inguinal region of New Zealand rabbits. At the third passage, cells were mixed with sodium alginate to prepare calcium alginate gel, and the cells were assigned into two-group cultivates in 24 orifice plates. ADSCs in the control group were treated with DMEM/F-12 medium supplemented with 10^−2^ mol/L β-glycerophosphate sodium, 10^−7^mol/L dexamethasone, 50 mg/L ascorbic acid, 0.1 % volume fraction of fetal bovine serum. ADSCs in the experimental group were incubated with the same medium as above, and in addition 1.5 µg/L CGRP was added. The cells proliferation and the mRNA expressions of collagen I and osteocalcin were detected by MTT and RT-PCR assays, respectively and alkaline phosphatase(ALP)and calcium concentration at different induction time were detected. The cell proliferation curves were S shaped. The OD values of experimental group were higher than those of control group at 1, 3, 5, 7, 14, and 21 days after osteogenic induction (*P* < 0.05). ALP and alizarin red stains of ADSCs were all positive, but golden round nodes became bigger and more in the experimental group compared with the control group after 2 weeks. At 7 and 14 days, collagen I and osteocalcin mRNA expression were greater in the experimental group than the control group. ALP and calcium concentration of experimental group were higher than that of control group at 1, 2, 3, 4 weeks after osteogenic induction (*P* < 0.05). Thus, these results show that the CGRP-induced ADSCs combined with calcium alginate gel to osteoblasts differentiation.

## Introduction

Calcitonin gene-related peptide (CGRP) is one of the most abundant neuropeptides. So far CGRP research has concentrated on its role on the nervous and the cardiovascular systems [1–3], but not on bone defect repair. Recent studies found that CGRP also plays an important role in bone repair and bone reconstruction. Hukkanen et al. (1993) found that CGRP-positive nerve fibers initially show degradation of periosteum and then quickly proliferate around the bone fracture in rats [4]. It is followed by the progress of the callus formation and bone remodeling along with the change in distribution and density. Aoki et al. (2004) confirmed that a large number of CGRP-positive nerve fibers were emerged in fiber granulation tissue, periosteum, and bone tissue during healing process of fracture in rats in an orderly manner with the degree of callus formation and hyperplasia of bone remodeling [5]. These hyperplasia sensory nerve fibers can release higher level of CGRP than the serum to participate in the normal process of bone healing. Li et al. (2007) also found that bone fracture can stimulate the generation of a large number of CGRP-positive nerve fibers around the injury in the rat model of tibial fracture which perhaps is a prerequisite for fracture healing and remodeling [6]. Hayashi et al. (2009) found that serum CGRP expression in rats markedly increased in early stage of spinal cord injury with fracture and the level is up to three times more than normal after 7 days of injury [7]. The difference between the level of CGRP expression after 3 and 7 days of injury and simple fracture was statistically significant (*P* < 0.01). Onuoha (2011) observed a change in plasma CGRP level (detected by ELISA method) within 24 h in patients after bone fracture. The plasma CGRP levels were significantly elevated in patients with bone fracture than the control group [8]. Sun Xiao-xin et al. (2009) reported that the CGRP expression in all cells which participated in the bone healing of patients group with fracture and complications of central nervous system injury was significantly higher than the simple fracture group at each time point in different stages of fracture healing through animal experiments. Also the formation and remodeling of callus at the fracture end happened earlier than that in the simple fracture group [9]. Ekelund et al. (1997) discovered that distribution density of CGRP-positive nerve fibers was positively related to the local amount of bone formation based on the study of distribution of the nerve fibers in the heterotopic ossification [10]. The CGRP-positive nerve fibers were sparse or absent in the nonunion human long bone shaft fracture. Furthermore, removal of sensory nerve terminals of CGRP-positive nerve fibers in the periosteum of rat tibia fracture, led to nonunion of fracture [11]. When the sciatic nerve was cut, the rat tibial fracture callus became bigger than normal but with low density and poor mechanical properties, and the callus grew without CGRP-positive nerve fibers [12]. In the rat model of spinal cord injury, formation of a large number of fibrous callus and cartilaginous callus was observed during early stage of tibial fractures, and CGRP proliferated apparently in the callus during this period. But CGRP decreased significantly after 2 weeks in the fracture callus, with significantly slow transformation of fibrous callus and cartilage callus into callus [13]. All these studies indicate that the CGRP and CGRP-positive nerve fibers play an important role in bone formation, bone repair, and bone reconstruction. CGRP receptors were also found to exist on the bone cell surface in vitro experiments [14]. But reports are rare about the role of CGRP to prompt adipose-derived stem cells into osteoblast differentiation. In the current study, we examined the effect of CGRP on the rabbit adipose-derived stem cells (ADSCs) to the proliferation and bone cell differentiation using alginate three-dimensional culture method in order to provide experimental basis for repair of bone defect with tissue engineering bone.

## Materials and Methods

### Design

Observational experiment.

### Time and Setting

These experiments were performed at the Central Laboratory of Affiliated Hospital of Xuzhou Medical College during 2012-07/2013-01.

### Materials

A total of two 3-month-old clean healthy New Zealand rabbits (A total of two clean healthy New Zealand rabbits of age 3 months), including male and female, weighing 2.5–3.5 kg, were provided by Experimental Animal Center of Xuzhou Medical College, license number: SCXK (Su) 2005-0005. The disposal of animal experimental process was in accordance with “Laboratory Animal Science Guidance” promulgated by the Ministry of Science and Technology in 2006 [15].

#### Key Reagents and Instruments

**Table:** 

Reagents and instruments	Company
DMEM/F-12 medium, fetal bovine serum (FBS)	Thermo, USA
β-glycerophosphate, dexamethasone, ascorbic acid, Recombinant human CGRP, sodium alginate	Sigma USA
Trypsin, collagenase type I, MTT cell proliferation and cytotoxicity detection kit	Beyotime institute of Biotechnology
Rabbit Anti-CD44/CD45	Beijing Biosynthesis Biotechnology CO., Ltd.
FITC-labeled Goat anti-rabbit IgG	Beijing ZSGB-BIO
ALP kit, ALP staining kit, Calcium assay kit	Nanjing Jiangcheng Bioengineering Institute
Total RNA extraction kit, RT kit, PCR reaction liquid	TIANGEN BIOTECH (Beijing) CO., LTD
Laminar flow hood	Suzhou Purification Equipment Co., LTD
Phase-contrast microscope	Olympus Optical Co., Ltd
Enzyme-linked immunosorbent assays	Toshiba (Japan) Co., Ltd.
Fluorescence microscopy	Nikon (Japan) Co., Ltd.
PCR Instrument	MJ Research Co., Ltd.
UV Gel imaging system	Alpha Innotech Co., Ltd.

### The Experimental Method

Culture and isolation of rabbit ADSCs [16, 17] 3-month-old New Zealand white rabbits, male or female, were anesthetized with 10 % chloral hydrate (2 mL kg^−1^) by ear vein injection. After skin preparation, and sterilization, an incision was made at median abdominal to the deep fascia, separate to the groin area on both sides of the bilateral inguinal to expose fat pad and remove it completely. Fat pad was put into 10-cm diameter petri dishes under sterile conditions, and outsourcing membranes were scavenged as much as possible of connective tissue and visible small blood vessels followed by 3–5 times wash with PBS to remove red blood cells. Tissue was cut into 1–2 mm^3^ blocks with ophthalmology shear, and 0.1 % I collagenase (3–5 times volume of the tissue) was added. Tissue was digested in a water bath at 37 °C and shaken for 60 min followed by an addition of an equal volume of DMEM/F-12 medium containing 10 % FBS to terminate the digestion. Tissue was then filtered with 200 mesh nylon filter, and centrifuged at 1200 rpm for 5 min. The centrifugal radius was 9 cm. The supernatant was discarded and the cells were suspended in DMEM/F-12 medium containing 10 % FBS. Cells were adjusted to the density of 1 × 109 L^−1^ and were then inoculated into 25 cm^2^ culture flasks containing basal medium. Monolayer cells were cultured in an incubator saturated with 5 % CO_2_ and constant temperature and humidity at 37 °C. After 24 h the culture bottle was shaken gently and examined under the microscope to make sure most of the cells are attached to the wall. Culture medium was then changed to remove non-adherent cells for the first time. Culture medium was changed once every 3 days, and the cell growth condition was observed under the inversion microscope. When the cell coverage was up to 80–90 %, the cells were purified and passaged for the first time.

### Identification of Rabbit ADSCs [18, 19]

Detection of marker on stem cell surface: the third-generation cells were inoculated in the preset coverslips in 6-well plates at 1 × 10^6^/mL, and 1.5 mL DMEM/F-12 medium containing 10 % FBS was added to each well. Cell plates were cultured at 37 °C, 5 % CO_2_ saturated humidity incubator until the cells were at 80–90 % of confluence. Cells were washed 3 times with pre-warm PBS, for 5 min each time, and fixed in 4 % paraformaldehyde for 30 min. Cells were again washed with PBS three times, for 5 min each time. Triton X-100 (0.2 %) was added for 2–5 min and then washed with PBS three times, for 5 min each time. Binding was blocked by 5 % goat serum and incubated at room temperature for 30 min. Blocking solution was discarded and the mouse anti-rabbit CD44, CD45 antibody reaction liquid was added, and incubated overnight at 4 °C. Antibody reaction liquid was removed and cells were washed with PBS three times, for 5 min each time. FITC-labeled anti-FITC antibody was added and incubated at 37 °C for 30 min followed by washing with PBS three times, for 5 min each time, and mounted with 95 % glycerol. Staining was observed under a fluorescence microscope.

At the same time, third-generation cells were inoculated in the preset coverslips in 6-well plates with 1 × 10^6^/mL and divided into two groups: bone-induced DMEM/F-12 medium containing 10 % FBS, 10^−2^ mol/L beta glycerophosphate, 10^−7^mol/L dexamethasone, and 50 mg/L ascorbic acid was added to the control group. Whereas 1.5 µg/L CGRP was added on the basis for induced culture in the experimental group.

### Alkaline Phosphatase Staining

After 2 weeks of induction, glass coverslips were taken from the experimental and the control group of 6-well plates and assay was continued in accordance with the instructions of alkaline phosphatase staining kit.

### Alizarin Red Staining

After 2 weeks of induction, cover glass from the experimental and the control groups of 6-well plates were fixed with 4 % paraformaldehyde for 30 min. 1 ml of 2 % alizarin red staining solution was added after cells were rinsed with PBS and incubated at room temperature for 20 min, observed under inverted phase microscope, and pictures were taken.

### Preparation of Composite Carrier and Experimental Groups

Sodium alginate (12 g/L) was added to the third generation of monolayer culture cells (2 ml 1 × 10^8^ L^−1^). The mixture solution was added to 24-well plates containing 1 ml 10^2 ^mmol/L CaCl_2_ in each well with straw (50 µl/per drop), about 2500 cells/per drop, and 200 µl of mixture was added to each well and incubated at 37 °C. After 10 min wait at room temperature to form the gel, washed 3 times with PBS, and divided into two groups: bone-induced DMEM/F-12 medium containing 10 % FBS, 10^−2^ mol/L β- glycerophosphate, 10^−7^mol/L dexamethasone, and 50 mg/L ascorbic acid was added to the control group. Whereas 1.5 µg/L CGRP was added on the basis for induced culture in the experimental group.

### Detection of ADSCs Proliferation Induced by MTT Method

Osteogenic cells in each group were removed after 1, 3, 5, 7, 14, and 21 days of culture, respectively (6 wells per each group). Composite carrier was dissolved with 55 mmol/L sodium citrate. Cells were then collected by centrifugation and added to the 96-well plates. To each well, 200 µL DMEM/F-12 medium (containing 10 % FBS and double antibiotics) and 20 µL MTT solution (5 mg/mL) was added and incubated at 37 °C for 4 h. Supernatant was aspirated out and discarded. DMSO (150 µL) was then added to each well and shaken for 10 min. Optical density (OD) of each well was tested in the ELISA tester at the wavelength of 490 nm. Growth curves with mean of 6-well OD values as the ordinate and time as abscissa were drawn.

### Detection of Expression of Type I Collagen and Osteocalcin mRNA by RT-PCR

Cultures were terminated on 7 and 14 days after osteogenic induction. Composite carrier was dissolved with 55 mmol/L sodium citrate and cells were then collected by centrifugation. Cells were lysed by Trizol and total cellular RNA was extracted. Purity of RNA was identified by reverse transcription to cDNA using RT RNA kit. The first strand of cDNA was then used as the template for PCR amplification.

- Sense primer of Osteocalcin: 5’-CATGAGAGCCCTCACA-3′.
- Antisense primers: 5′-AGAGCGACACCCTAGAC-3′.
- Type I collagen sense primer: 5’-GGCAAACATGGAAACCG-3′.
- Antisense primers: 5’-TCAAGGAAGGGCAAACG-3′.

PCR conditions: pre degeneration at 94 °C for 5 min, then denatured at 94 °C for 30 s. Annealed at 52 °C for 30 s. Extends at 72 °C for 1 min, a total of 30 cycles, and extension at 72 °C for 10 min. PCR product was confirmed by electrophoresis, DNA absorbance scan detection. The β-actin bands represent parameters for mRNA expression level.

### Detection of Alkaline Phosphatase Activity

Two groups of cells (4 wells for each group) were removed after 1, 2, 3, 4 weeks of inducement, respectively. Composite carrier was dissolved with 55 mmol/L sodium citrate and cells were then collected by centrifugation and added to the 96-well plates. TritonX-100 (0.2 ml 1 %) was added and incubated at 4 °C overnight, and assay was performed in accordance with the alkaline phosphatase test box instructions. Alkaline phosphatase concentration was expressed as king unit/100 mL.

### Detection of calcium ion concentration

Cells (4 wells for each group) were removed in each group after 1, 2, 3, 4 weeks of inducement respectively. Composite carrier was dissolved with 55 mmol/L sodium citrate and the cells were collected by centrifugation and added to the 96-well plates. TritonX-100 (0.2 mL1 %) was added and incubated at 4 °C overnight, and the assay was run in accordance with the calcium ion detection kit instructions. The concentration of calcium ion is represented by mmol/L.

### Main Outcome Indicator

① Expression of CD44, CD45 on ADSCs surface was detected by immunofluorescence staining. ② The adipose-derived stem cell morphology observation and the proliferation curve. ③ Detection of ADSCs osteogenic differentiation by alkaline phosphatase staining and alizarin red staining. ④ The expression of osteocalcin and type I collagenase mRNA. ⑤ The osteoblast alkaline phosphatase activity and calcium ion concentration.

### The Design, Implementation, and Evaluation

Design and evaluation by the first and second author. Intervention is carried out by the first, third, fourth, and fifth authors. Both of them are been trained formally.

### Statistical Analysis

The analysis was performed using the SPSS16.0 statistical software package. Data are shown as mean ± standard deviation (*X* ± *s*). The groups were compared using the paired sample *t* test and single factor analysis of variance. Two groups were compared with SNK test. *P* < 0.05 was considered statistically significant.

## Results

### Observation of Morphology of ADSCs

**Fig. 1:**
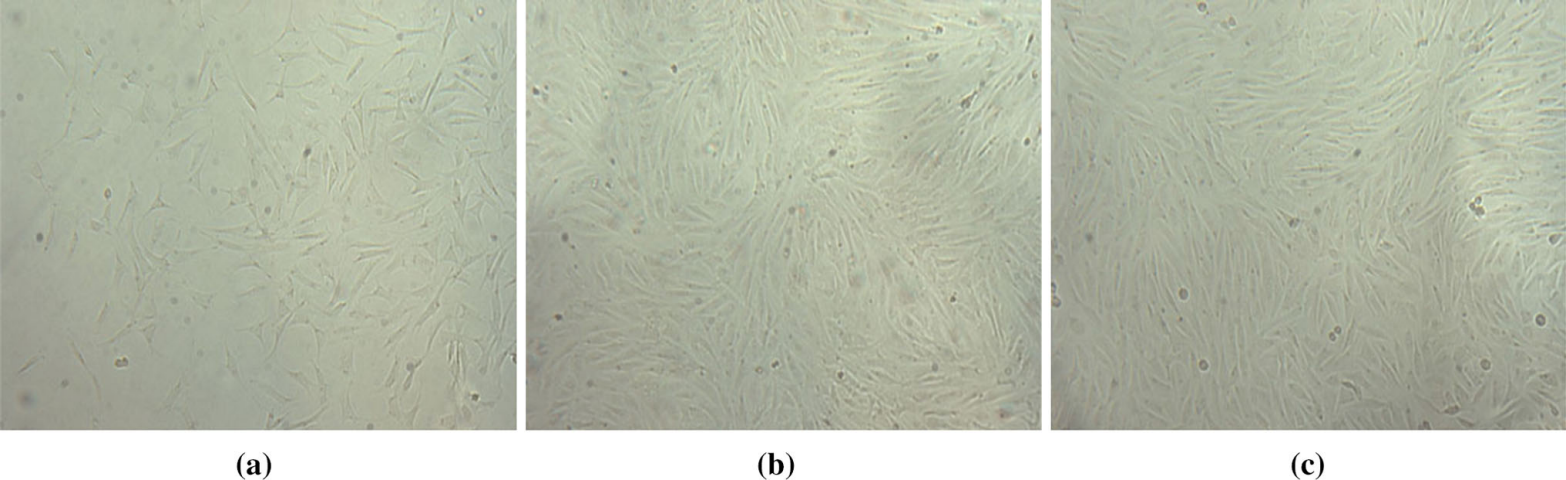
Morphology of ADSCs (Inverted contrast-phase microscope ×100). **a** Primary ADSCs cultured 24 h. Most of adherent cells were observed as fusiform, polygonal after the first liquid change to remove non-adherent cells **b** Primary ADSCs cultured 6 days. The morphology of cells was typical fusiform. The confluence was 90 % after 6 days in a “vortex” arrangement. **c** The ADSCs at passage 3 cultured 3 days. The morphology of the third-generation cells was typical fusiform after 3 days in a “vortex” arrangement

Cells were small and round at the beginning of isolation. Adherence happened after 24–48 h of culture. Non-adherent cells were round or spherical red blood cells. Most of adherent cells were observed as fusiform, polygonal after the first liquid change to remove non-adherent cells (Fig. 1a). With the extension of incubation time and the amount of cells, morphology of cells was typically fusiform in a colony and of nodular growth type. The confluence was 80–90 % after 5–7 days in a “vortex” arrangement (Fig. 1b). To accelerate the speed of cell growth after subculture, 3–4 days can be passaged, and long-term stable cultured (Fig. 1c).

### Identification of ADSCs

**Fig. 2:**
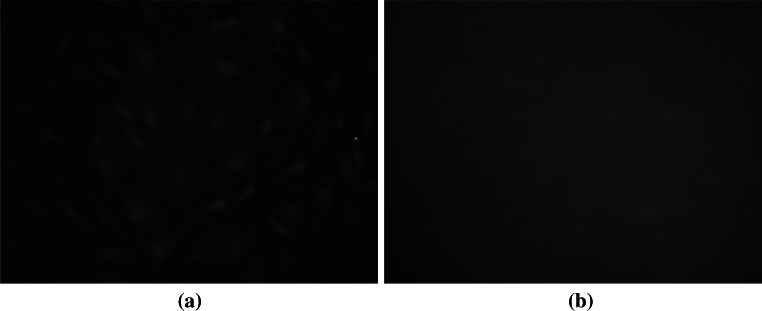
Identification of ADSCs. **a** Positive CD44 immunofluorescence staining (Fluorescence microscope ×200). **b** Negative CD45 immunofluorescence staining (Fluorescence microscope ×200)

Detection of immunofluorescence staining of ADSCs surface molecule method showed: CD44 cell surface antigen is positive (Fig. 2a), showing that the cells are mesenchymal stem cells. Whereas CD45 cell surface antigen was negative (Fig. 2b), which proved that the detection cell sources are not in blood circulation from stem cells.

### Identification of Bone

**Fig. 3:**
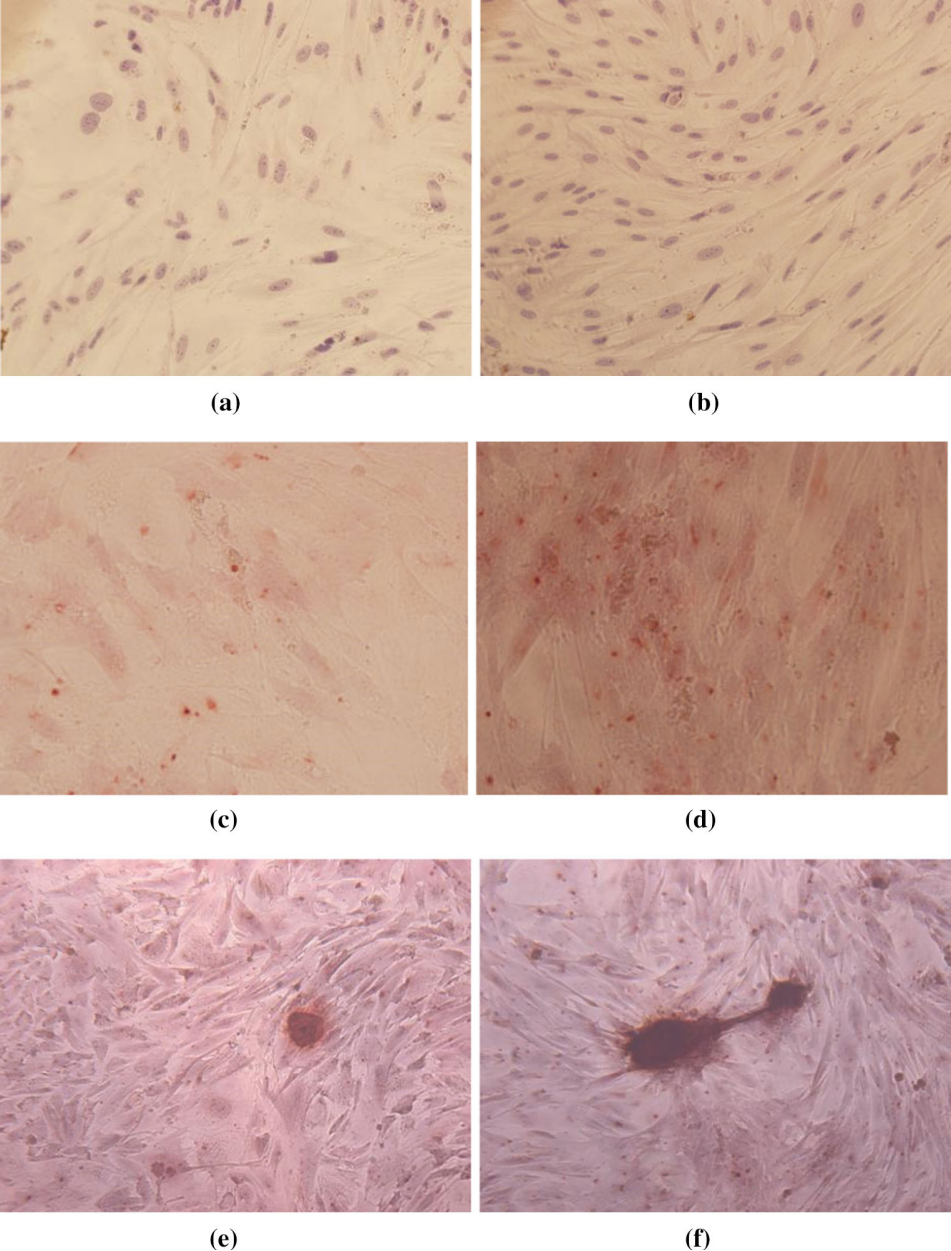
The results of alkaline phosphatase and alizarin red staining after ADSCs cultured for 14 days (Inverted contrast-phase microscope ×200). **a** The alkaline phosphatase staining of control group. ALP staining in control group (×200). **b** The alkaline phosphatase staining of experimental group. ALP staining in experimental group (×200). **c** The alizarin red staining of control group. Alizarin red staining in control group (×200). **d** The alizarin red staining of experimental group. Alizarin red staining in experimental group (×200). **e** The calcium nodules of control group. Calcified nodules in control group (×200). **f** The calcium nodules of experimental group. Calcified nodules in experimental group (×200)

Cells of both groups were alkaline phosphatase staining and alizarin red staining positive after 2 weeks of induction (Fig. 3a–f), but the calcified nodules of experimental group were significantly more and bigger than those of the control group.

### Detection of the Proliferation Activity of ADSCs After Osteogenic Induction by MTT Method

**Fig. 4:**
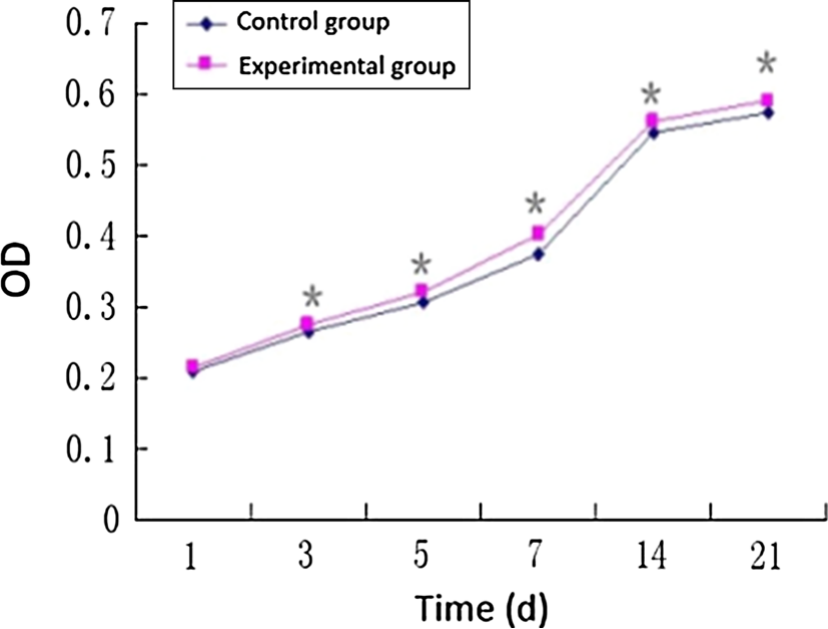
The cells proliferation of both groups (*Compared with control group,*P* < 0.05,*n* = 6)

MTT detection shows that OD values were increased with the prolongation of time of cells in both groups. But except for the first day, the differences in the different time points were statistically significant in the CGRP-induced group and the control group (*P* < 0.05). And change in different induced phases was statistically significant in CGRP-induced group (*P* < 0.05), showing that the CGRP promotes osteogenic differentiation of ADSCs (Fig. 4).

### Detection of Type I Collagen and Osteocalcin mRNA Expression by RT-PCR

**Fig. 5:**
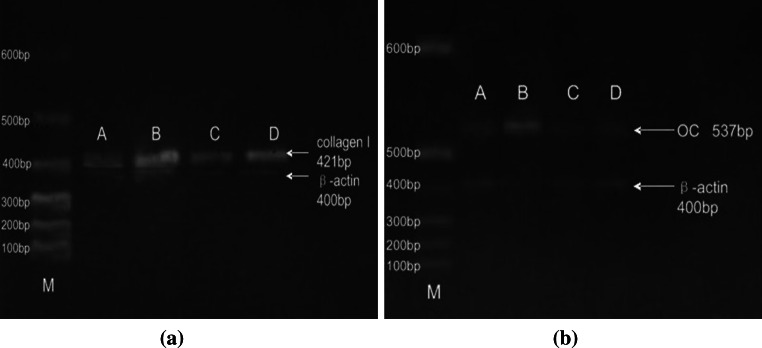
Reverse transcriptase-polymerase chain reaction (RT-PCR) results of collagen I and osteocalcin mRNA between the two groups **a** collagen I **b** Oc. **a** The detection results of RT-PCR of cell type I collagen mRNA expression. **b** The detection results of RT-PCR of cells osteocalcin mRNA expression

Results of RT-PCR show that the expression of type I collagen and osteocalcin mRNA in the experimental group of 7 and 14 days osteogenesis inducement was better than those in the control group (Fig. 5a, b).

### Detection of Alkaline Phosphatase Concentration

ALP concentrations showed differences between the experimental group and the control group. The ALP concentrations at each time point in experimental group were higher than that of the control group (*P* < 0.05, *n* = 4). The change trend of ALP concentration for both groups was also different. The change was even more significant in experimental group. ALP concentration declined gradually in 2 weeks after the peak in both groups (Table 1).

**Table 1:** Alkaline phosphatase expression in both groups after induction ($\bar{x}$± s, King unit/100 ml)

Group	n	1 week	2 weeks	3 weeks	4 weeks
Experimental group	4	0.8141 ± 0.0780*,^#^	1.6916 ± 0.1333*,^#^	1.0666 ± 0.3879*,^#^	0.8790 ± 0.1170*,^#^
Control group	4	0.3614 ± 0.0239	0.5865 ± 0.0840	0.4406 ± 0.1373	0.3876 ± 0.0557

* Calcium concentration of experimental group was distinct in different tendency under different induction (*P* < 0.05)

^#^ Compared with control group, *P* < 0.05

### Detection of Calcium Ion Concentration

Difference in Calcium ion concentration existed between the experimental and control groups. The calcium concentration at each time point in experimental group was higher than the control group (*P* < 0.05, *n* = 4). The change trend of calcium ion concentration of these groups was also different. The trends were similar for the first and second week in both groups. Variation amplitude was significantly higher in third and fourth week for experimental group compared with control group (Table 2).

**Table 2:** Calcium concentration of two groups after induction ($\bar{x}$± s,mmol/L)

Group	*n*	1 week	2 weeks	3 weeks	4 weeks
Experimental group	4	1.1883 ± 0.1365*,^#^	1.8814 ± 0.0870*,^#^	3.2878 ± 0.2354*,^#^	5.5449 ± 0.5088*,^#^
Control group	4	0.5431 ± 0.1472	1.1367 ± 0.0602	1.5740 ± 0.0919	2.6086 ± 0.0976

* Calcium concentration of experimental group was distinct in different tendency under different induction (*P* < 0.05)

^#^ Compared with control group, *P* < 0.05

## Discussion

Repairing bone defect caused by various factors such as infection, trauma, tumor has always been one of the difficulties in the Department of Orthopedics. The rise and rapid development of tissue engineering technology brings a new therapeutic approach for repair and reconstruction of bone defects. Bone tissue engineering, and transgenic engineering bone [20–23], research has also made some progress in bone defect repairing. Adipose-derived stem cells (ADSCs) is a kind of newly discovered adult stem cells, are becoming a hot research topic in tissue engineering, regenerative medicine, and other fields because of the advantage of being a rich source, convenience, small injury to the body, fast proliferation, multipotential differentiation, and higher safety [24]. Tissue engineering technology mainly includes the seed cells, biological active factor, and carrier material. ADSCs as a new source of seed cells have become the focus of the study of bone and cartilage tissue engineering. Calcium alginate gel as carrier with three-dimensional structure, good biocompatibility, biodegradable, hydrophilic, good cell adsorption, and plasticity, could be in favor of the diffusion between nutrients and cell contact [25]. It has already been approved by FDA and widely applied in pharmaceutical dosage forms, food industry, wound dressing, and bone and cartilage tissue engineering.

CGRP as one of the most abundant neuropeptides is a biologically active polypeptide found during the methods of DNA reorganization and molecular biology technology in 1983 by Rosenfeld et al. (1983). Morris et al. (1984) extracted CGRP from human medullary thyroid carcinoma tissue for the first time in which it was confirmed that the peptide exists in human bodies [26, 27]. Nowadays it has been reported that human, rat, mouse, rabbit, and other spinal animal body contains CGRP [28]. CGRP is a kind of multifunctional peptide hormone, divided into two kinds of subtypes CGRP-1 and CGRP-2, and known as the most powerful vasodilator substance [28], which has been appreciated extensively because of its extensive biological effects. CGRP plays an important role in the formation of bone which can promote bone formation and inhibit bone absorption [29, 30]. Years of studies have proved that CGRP receptor exists in four kinds of cells which are directly related to metabolism and bone marrow, including stromal cells, osteoblasts, and bone marrow macrophages and osteoclasts [29]. Jimenez-Andrade et al. (2010) found the CGRP receptor mRNA expression on the osteoblast surface using RT-PCR method, confirming the presence of CGRP receptor in osteoblasts [31]. This study found that the CGRP regulation of osteogenic cells is based on its regulation of cell growth factor such as Ca^2+^, cAMP, PCK, IGF, etc [32].

In this study, we used CGRP to promote the differentiation of adipose-derived stem cells into osteoblast which is in the three-dimensional culture conditions of calcium alginate gel. Two groups of cells of alkaline phosphatase and the calcified nodules staining were positive 2 weeks after induction. Analyzing the change in the concentration of alkaline phosphatase and calcium ion 1, 2, 3, 4 weeks after the induction, we found that the concentration of alkaline phosphatase increased from the first to the second week, and decreased steadily thereafter. However, the concentration of calcium increased in all the 4 weeks. CGRP in induction group was significantly higher than that of control group. Compared with the control group, calcified nodules in the experimental group were increased and with large volume. Expression of osteoblast marker of type I collagen and RT-PCR detection of osteocalcin mRNA 7, 14 days after inducement in the experimental group was better than the control group. This proves the existence of differentiation from the adipose-derived stem cells to bone cells, and also that CGRP can promote the differentiation of adipose-derived stem cells into osteoblasts at the same time.

In conclusion, this experiment successfully induced rabbit adipose-derived stem cells into osteoblast differentiation in vitro using recombinant human CGRP in three-dimensional culture conditions, but whether the osteoblasts have the same biological properties in vivo and repair the bone defect after induction remains unclear, and needs further animal experiments.
